# No Evidence for the Effect of MHC on Male Mating Success in the Brown Bear

**DOI:** 10.1371/journal.pone.0113414

**Published:** 2014-12-03

**Authors:** Katarzyna Kuduk, Wieslaw Babik, Eva Bellemain, Alice Valentini, Andreas Zedrosser, Pierre Taberlet, Jonas Kindberg, Jon E. Swenson, Jacek Radwan

**Affiliations:** 1 Institute of Environmental Sciences, Jagiellonian University, ul. Gronostajowa 7, 30-387, Kraków, Poland; 2 SPYGEN, Savoie Technolac - Bât. Koala 17, rue du Lac Saint-André - BP 274, 73375, Le Bourget-du-Lac Cedex, France; 3 Department of Ecology and Natural Resource Management, Norwegian University of Life Sciences, NO-1432, Ås, Norway; 4 Institute for Wildlife Biology and Game Management, University of Natural Resources and Life Sciences, AT-1180, Vienna, Austria; 5 CNRS, LECA, F-38000, Grenoble, France; 6 Université Grenoble Alpes, LECA, F-38000, Grenoble, France; 7 Department of Wildlife, Fish and Environmental Studies, Swedish University of Agricultural Sciences, SE-901 83, Umeå, Sweden; 8 Norwegian Institute for Nature Research, NO-7485, Trondheim, Norway; Universidade de São paulo, Brazil

## Abstract

Mate choice is thought to contribute to the maintenance of the spectacularly high polymorphism of the Major Histocompatibility Complex (MHC) genes, along with balancing selection from parasites, but the relative contribution of the former mechanism is debated. Here, we investigated the association between male MHC genotype and mating success in the brown bear. We analysed fragments of sequences coding for the peptide-binding region of the highly polymorphic MHC class I and class II DRB genes, while controlling for genome-wide effects using a panel of 18 microsatellite markers. Male mating success did not depend on the number of alleles shared with the female or amino-acid distance between potential mates at either locus. Furthermore, we found no indication of female mating preferences for MHC similarity being contingent on the number of alleles the females carried. Finally, we found no significant association between the number of MHC alleles a male carried and his mating success. Thus, our results provided no support for the role of mate choice in shaping MHC polymorphism in the brown bear.

## Introduction

MHC genes comprise the most polymorphic gene family in vertebrates, with hundreds of alleles found in many vertebrate species (reviewed in: [1], [2]–[4]). Their protein products are responsible for binding oligopeptides (antigens), products of protein degradation, and presenting them to lymphocytes, a mechanism serving the recognition of self from non-self [5]. The high polymorphism of MHC genes is thought to result from their function in recognising pathogen assault. This view is supported by abundant evidence for associations of MHC alleles with resistance or susceptibility to parasites (in a broad sense including microorganisms) (reviewed in: [2], [3], [4], [6]) and an excess of non-synonymous substitutions in sequences coding for the peptide-binding region of MHC molecules [7]. Frequency-dependent selection, arising because the bearers of common alleles are more likely to be evaded by fast-evolving parasites [8]–[10], and the advantage of heterozygotes able to present a wider range of pathogen-derived peptides [11], [12], are considered major mechanisms responsible for the maintenance of MHC polymorphism.

Apart from parasites, sexual preferences for MHC-dissimilar mates might contribute to high polymorphism of MHC genes. The first evidence for such preferences was provided by studies of congenic strains of laboratory mice by Yamazaki and colleagues [13], [14], who recognized that disassortative mating with respect to MHC loci could help to explain the high MHC polymorphism observed in wild populations. Population-genetic [15] and simulation [16] models confirmed that disassortative mating with respect to MHC type indeed can lead to the maintenance of polymorphism. Preferences for MHC-dissimilar mates may arise because they prevent mating with relatives or because they lead to production of MHC-heterozygous progeny with a superior resistance to parasites (reviewed by [17], [18]). MHC-based sexual preferences now have been demonstrated in many vertebrate species, including fish [19], [20]; reptiles [21], birds [22], [23], and mammals [24]–[27], although negative results are not uncommon (eg. [28], [29]–[33]).

Although there is evidence in support of the role of both parasites and mate choice in maintaining MHC polymorphism, their relative importance is not well understood. In a recent meta-analysis on mammals, Winternitz et al. [34] found some evidence for MHC diversity being associated with parasite richness in bats and ungulates, but among carnivores the relationship was reversed. Interestingly, Winternitz et al. have also found a correlation between MHC diversity and testis size, which they used as a proxy for the strength of sexual selection. This suggested to the authors that mate choice may play a major role in maintaining MHC polymorphism.

MHC class I genes are expressed in all nucleated cells and present antigens derived mostly from intracellular parasites, whereas MHC class II genes are expressed in specialised antigen-presenting cells, such as macrophages, and present mostly antigens of extracellular parasites. The peptide-binding groove of class I molecules is formed by α1 and α2 chains encoded by the second and third exon of the gene, whereas the class II peptide-binding groove is formed by α and β chains encoded by second exons of separate A and B genes [5]. The relative roles of MHC I and MHC II in mediating mate choice are not clear. Because of ubiquity of expression of MHC I molecules, they could be expected to be the main source of MHC ligands, which are known to play a role in MHC genotype perception in mice [35]. However, MHC II may shape the composition of bacterial communities carried by a host, which have been shown to affect individual odour specificity [36]. Whereas there is evidence for the role of MHC I in mate choice in mice [35], [37], a recent study investigating the relative roles of both MHC classes in mate choice in blue petrels showed that only class II plays a role [23].

Here, we investigated whether MHC-disassortative mating occurs in the brown bear (*Ursus arctos*).The Scandinavian population we studied is highly polymorphic at MHC I, whereas at class II, only DRB genes were highly polymorphic [38]. Consequently, we investigated whether male mating success was dependent on his MHC similarity to a female at either class I or class II DRB.

In the brown bear, both MHC I (comprising 3 expressed loci), and class II DRB genes (2 loci) have been shown to be under historical positive selection, but the excess of non-synonymous substitutions was particularly strong at DRB [38], a pattern also present in canids [39]. This suggests that this gene may either be under particularly strong selection due to parasites, or that it may be particularly important in mate choice.

Apart from the similarity to female MHC, we also investigated whether male mating success depends on the number of MHC I or DRB alleles carried by a male. If heterozygosity at MHC is associated with higher resistance to parasites, males that are MHC heterozygous (or carrying more alleles in multilocus systems) can be more attractive to females, as reported for rhesus macaques [29] and mice [40]. Similarly, in the fat-tailed dwarf lemur, where the DRB locus is duplicated, males carrying more alleles achieved higher reproductive success. However, in species in which MHC loci occur in several copies, both positive [41] and quadratic [42], [43] relationships between the number of alleles and parasite resistance have been reported. A quadratic relationship may arise because of a trade-off associated with expressing many MHC alleles; on the one hand, it should allow binding a wide range of antigens, but on the other hand, it may limit the repertoire of T-cell receptors (TCRs), due to more intense negative selection. TCRs bind MHC-antigen complexes with high specificity and their high diversity is required for the immune system to recognise a wide range of pathogens. Hence, an optimal, rather than a maximal, number of alleles may give the most effective immune response [44], [45], and consequently intermediate numbers of alleles should be associated with the highest reproductive success. Therefore, our tests considered both linear and non-linear relationships between the number of alleles and male mating success.

Furthermore, if the intermediate number of alleles is favoured, female preferences for male MHC type may be contingent on their own MHC type. Thus, females with a low number of MHC alleles should prefer males with many MHC alleles and vice versa, as documented in sticklebacks [46]. Here, we also investigated whether a similar relationship occurs in the brown bear.

### Study species

The brown bear is characterised by a promiscuous mating system. During the mating season, male and female brown bears roam to mate and remain together for a few hours to several days, and both males and females mate promiscuously [47]. Female bears are induced ovulators [47], which may provide females with more control over the paternity of their offspring than with spontaneous ovulation [48]. Females give birth to 1–4 small cubs in January, while still hibernating in dens. Young bears receive extended maternal care, staying with the mother for 1.5–2.5 years in the studied populations [49], [50]. Cub mortality averages 35% annually in the southern study area and 4% in the northern study area [51].

## Methods

### Study area and sampling

Samples analyzed in the present study originated from brown bear populations sampled within the Scandinavian Brown Bear Research Project. We used data from two large areas in Sweden. The southern study area consisted of ∼13,000 km^2^ centered on 61°N, 15°E and the northern area consisted of ∼8,000 km^2^ centered on 67°N, 17°E. All captures were approved by the Swedish Ethical Committee on Animal Research in Uppsala (application numbers C212/9 and C47/9) and the Swedish Environmental Protection Agency (Dnr 412-7327-09 Nv). The capture and sampling protocols are described thoroughly in Arnemo et al. 2011; http://www.bearproject.info/uploads/publications/2011%20Biomedical%20Protocols%20Carnivores.pdf. Genetic samples were obtained from yearlings captured with their mothers, because we did not capture young-of-the-year for ethical reasons [52]. This means that we did not obtain samples from young that died during their first year of life. Cub mortality is, however, mostly explained by sexually selected infanticide. Indeed cub mortality is much higher in the southern area (35%), where male turnover is high due to hunting, but usually (68% of the cases) results in the loss of whole litters [51], so such cases were not entered into our dataset. In the more socially-stable northern population, mortality is lower (4%). Thus overall, cub mortality had little effect on paternity assignment. The study was carried out on both private and public land in Sweden. The Swedish Environmental Protection Agency's capture permit is general and valid for all of Sweden, including public and private lands. Thus, prior permission from private landowners was not required. Field work involving helicopters and snowmobiles in national parks and other protected areas was approved for biennial periods by the County Administrative Boards in Norrbotten and Dalarna countries. Details on sampling and genomic DNA (gDNA) extraction can be found in Bellemain et al. [47].

### Genotyping

We used two families of classical MHC genes that were found to be polymorphic in a previous study [38]; MHC class I, consisting of at least 3 expressed loci, and class II DRB, consisting of 2 loci. We amplified large fragments of exons coding for peptide-binding regions of MHC molecules; 228 bp of MHC class I 2^nd^ exon and 192 bp of class II DRB 2^nd^ exon, using primer pairs URS_1_F – URS_1_R (MHC I) and URS_DRB_F3 – URS_DRB_R, which were designed specifically for the brown bear by Kuduk et al. [38].The 2^nd^ exon of the DRB reflects most of the functional variation of the peptide-binding groove of this MHC II molecule, as its second half is coded by a DRA gene characterised by low polymorphism across mammals [1]. In MHC I molecules, the second half of the groove is formed by a polymorphic 3^rd^ exon. However, MHC haplotypes extend up to 1 Mb from the centre of the gene, such that sequence of one exon is likely to carry sufficient information on allelic identity [53]. Amplicons were sequenced using 454 technology and genotyping-by-sequencing was performed as described in Kuduk et al. [38]. The authors found 100% consistence of genotypes for two independent replicates for MHCI, and low (2.6%) genotyping error for DRB. For further analyses, we excluded MHC class I alleles that fell into two pseudogene clusters [38].

For each female, we calculated MHC similarity to a set of her potential mates. The potential mates were defined as males that were recorded within 40 km from the home range of the female, as this is the distance below which 95% of actual reproduction events occur (see [47] for details). On average, there were 11.1 potential mates/female (range 2–25).

We used two measures of MHC similarity. The first was the proportion of shared alleles between males and females, i.e. the number of alleles shared divided by the total number of alleles found in a given male and female pair. The second was the mean amino-acid distance (henceforth AA distance), between female and male alleles. We calculated the AA distance for each possible male-female pair of alleles using MEGA 5.1. The distances were calculated separately for MHCI and DRB loci. We also calculated overall similarity as the mean of MHC I and DRB values. We also attempted to group alleles into supertypes [54] with DAPC using adagenet package in R [55], [56]; however, the find.cluster function did not indicated that the optimal number of supertypes was lower than the number of alleles for both MHCI and DRB, implying that grouping alleles into supertypes would not be justified.

We used estimates of paternity, relatedness, and heterozygosity, obtained by Bellemain et al. [47] on the basis of a set of 18 microsatellite loci, to discriminate between successful and unsuccessful males and to control for genome-wide similarity between mating partners and the genome-wide heterozygosity of males. Of the 114 males that sired progeny (henceforth: successful males), two shared paternity with other males, and the remaining males sired all of the cubs in the litter. The final dataset consisted of genotypes for 912 female–potential mate pairs, 114 of which were female-successful mate pairs, and 788 were female-unsuccessful potential mate pairs.

### Statistics

To analyse the effect of MHC similarity on the probability that a male sired the young of a given female, we used generalized linear mixed models implemented in the MCMCglmm package in R [57], with paternity as a binomial response variable, MHC similarity (either AA distance or proportion of shared alleles) as a predictor, relatedness, and body size as covariates, and female id, male id, and year as random factors. Similar models were run to check whether the number of MHC alleles affected male paternity, but with male multi-locus heterozygosity at 18 microsatellite alleles as a covariate. We also entered a quadratic relationship between the number of alleles possessed by a male and mating success to test if males with an intermediate number of alleles achieved the highest mating success.

In order to investigate if the probability for males with different numbers of alleles to obtain paternity depended on a number of alleles possessed by the female, we calculated the relative MHC diversity of a sire as the difference between the number of alleles he carried and the average number of alleles possessed by unsuccessful males that were potential mates of a given female in a given year. We also calculated similar relative measures of genetic similarity (proportion of shared alleles or AA distance). We then ran MCMCglmm with relative MHC diversity or similarity of a sire as a dependent variable, the number of alleles carried by a female as a predictor, and female id as a random factor.

## Results

We found 43 MHC class I alleles, including 11 alleles belonging to pseudogene clusters and 4 alleles from putative non-classical loci. Six alleles have not been reported previously (GenBank accession no KM242064- KM24206). Only 28 putatively expressed alleles from classical MHC I genes were included in our analyses. There were 2–7 such alleles per individual. For DRB, we found 17 alleles in our sample, including 1 allele that had not been reported previously (File S1). Individual bears carried 2–4 DRB alleles. The proportion of alleles shared between females and potential mates ranged between 0 and 33% for MHC I and 0 and 50% for MHC II DRB. AA distances ranged between 0.100 and 0.189 for MHCI and 0.037 and 0.239 for DRB. Mean proportions of MHC I alleles shared between a female and her potential partners are shown in Table 1. At both MHC I and DRB, there was no significant difference between successful and unsuccessful males in the number of alleles shared with females (Table S1 in File S1), P_MCMC_ = 0.764 for MHC I and P_MCMC_ = 0.324 for DRB). Similarly, we detected no significant effect when we used the mean proportion of alleles shared for MHC I and DRB as a predictor (P_MCMC_ = 0.480).

**Table 1:** Means and standard errors of MHC genetic similarity (proportion of shared alleles and amino-acid distance, see methods) at MHC class I and class II DRB loci between female brown bears, successful males (sires), and unsuccessful potential mates in Scandinavia.

	successful (mean ± SE)	unsuccessful (mean ± SE)
Proportion shared MHC I	0.109±0.005	0.117±0.004
Proportion shared DRB	0.113±0.009	0.122±0.004
AA distance MHC I	0.158±0.001	0.156±0.001
AA distance DRB	0.159±0.002	0.157±0.001
Number of MHC I alleles	3.579±0.082	3.505±0.031
Number of DRB alleles	4.613±0.124	4.465±0.046

Genetic similarity to the females, measured as mean AA distance, did not differ significantly between successful and unsuccessful males at either MHC I or DRB (Table 1, Table S2 in File S1, P_MCMC_ = 0.864 for MHC I and P_MCMC_ = 0.172 for DRB). Likewise, the mean AA distance for MHCI and DRB did not differ between successful and unsuccessful males (P_MCMC_ = 0.466).

The number of alleles carried by a female was not significantly associated with the relative MHC diversity of her mate (Table S3 in File S1). This was true for MHC I (P_MCMC_ = 0.746), DRB (P_MCMC_ = 0.084), and for MHC I and DRB combined (P_MCMC_ = 0.848). Likewise, the number of alleles carried by a female was not related to the female's relative MHC similarity to the sire (P_MCMC_>0.304 for all tests, Table S3 in File S1).

The number of alleles carried by males did not have an effect on their mating success for MHCI, DRB, or their sums (Table S3 in File S1, P_MCMC_>0.2 in all cases). However, a male's mating success depended significantly on his genome-wide heterozygosity (Table S4), as documented earlier [47].

## Discussion

There are two possible pathways by which the MHC genotype may affect male mating success. Firstly, genetic similarity between the male and female may affect mating probability (eg. [21], [23], [24]), and secondly, a male's MHC type may affect his mating success independently of the female's MHC genotype (eg. [25], [29], [40], [58]). Our results did not provide support for either of these mechanisms.

Regarding the first mechanism, previous work found no evidence for preferences for unrelated males [47]. Nevertheless, preferences for MHC-dissimilar partners could evolve independently of inbreeding avoidance, as such preferences would increase MHC heterozygosity, and consequently parasite resistance, of progeny. However, we did not find any evidence that males sharing fewer alleles with the female, or carrying alleles with lower amino-acid sequence similarity to the female, were more likely to sire her progeny. Huchard et al. [59] found that disassortative mating with respect to MHC class II in grey mouse lemurs is locus specific, with the DRB, but not the DQB, locus showing a significant effect. They suggested that this may be due to stronger selection acting on DRB, as revealed by sequence evolution, but reversed causality cannot in fact be excluded. DRB is under particularly strong selection also in the brown bear [38], and we have hypothesized that this may be partly due to selection from mating preferences. However, we found no evidence for MHC-based mating preferences for DRB, nor for MHC I. This conclusion also holds for their joint effect. Whereas the assessment of the similarity based on allele-sharing should be little affected by the fact that only one of the two polymorphic exons coding for the peptide-binding groove of MHC I molecule were sequenced (see methods for justification), amino-acid similarity at MHC I could have been less precise than that at DRB. However, consistence of negative results across different measures and genes strongly suggests that mate choice is not based on MHC similarity in the brown bear.

The lack of significant relationships could be due to female preferences for MHC similarity being contingent on the numbers of alleles she carried, such that females with many alleles might prefer males that are more MHC similar, as documented for sticklebacks [46]. However, we have found that the number of alleles that female brown bears carried was not related to their preferences for MHC similarity. Although MHC-based mate preferences have been demonstrated in several species of mammals, including mice [24], bank voles [26], and humans [27], no evidence for such preferences were found in Soay sheep [28], rhesus macaques [29], or Malagasy giant jumping rats [31]. Our study thus adds another example illustrating the fact that MHC-based preferences are not universal, even among mammals, a group in which olfactory cues play an important role [60]. Whereas there are signatures of positive selection on MHC in the brown bear, particularly strong at the DRB locus, it is possible that they are due to selection for particular alleles, rather than MHC heterozygosity (see [6] for the recent review of mechanisms). If MHC heterozygosity is not favoured by selection, evolution of preference for MHC-dissimilar mates is not expected to evolve, especially because another reason for MHC-disassortative mating, i.e. inbreeding avoidance, does not seem to take place in Scandinavian populations of brown bear [47]. Additionally, because of strong size dimorphism in the brown bear, males may coerce females to mate, thus decreasing the potential for female mate choice. Nevertheless, Bellemain et al. [47] reported that the first male to be observed with the female was not the father of her progeny in 68% of the cases. This suggests that females have a degree of control over mating and/or fertilization. Bellemain et al. [47] suggested that female brown bears may mate with multiple males as a strategy to avoid male infanticide, and use post-copulatory mechanisms to control paternity [61], [62]. However, female brown bears do not seem to use this strategy to select sperm from less related [47] or MHC-dissimilar males (this study). Similarly in house mice, where the mating system is also characterized by male infanticide, females do not increase offspring MHC diversity via multi-male mating [63], even though females are apparently able to discriminate MHC-based odour cues [17], [24].

Although our results indicate that a male's mating success did not depend on his MHC similarity to the female, we cannot exclude the role of MHC in maternal-fetal interactions, leading to the abortion of MHC-similar embryos [64], [65]. Unfortunately, we were not able to test for overrepresentation of non-shared alleles in progeny, because, due to co-amplification of duplicated genes, alleles could not be ascribed to loci, and thus the genotypic states could not be determined. This made it impossible for us to calculate expected and observed allele proportions in progeny. However, new methods based on ultra-deep sequencing have recently been developed that should enable inferring genotypic states for co-amplifying loci [66]. Thus, this hypothesis will hopefully be addressed in future research.

As to the second mechanism, we did not find a significant effect of the number of MHC alleles a male carries and his mating success. Such an association should arise if males carrying a large (or intermediate) number of alleles are more resistant to parasites [42], [67] and infection with parasites decreases male mating success [58], [68], [69]. Indeed, heterozygous males, or those with larger number of MHC alleles, have been shown to achieve higher reproductive success in several mammalian species [25], [29], [70]. In contrast, we found no effect of MHC allele copy number on male mating success, either in a linear or a quadratic form. This was true for both MHC I and DRB. Instead, we documented a significant, positive effect of genome-wide heterozygosity, as reported in an earlier study [47]. The lack of the effect of the number of MHC alleles may be due to infection status being associated with specific MHC alleles, rather than their number (eg. [58], [59], [64], [71]).

In conclusion, our study provided no evidence for an effect of MHC on male mating success in the brown bear. This negative effect is unlikely to result from the lack of statistical power, as we did detect a significant effect of genome-wide heterozygosity. Although we cannot exclude the role of maternal-fetal interaction as a source of selection on MHC, signatures of positive selection that are apparent in brown bear MHCI and DRB sequences [38] are likely to be due to host-parasite coevolution. The association between MHC and infection in brown bear awaits future investigation.

## Supporting Information

File S1 **Supporting files.** Table S1, The effect the proportion of shared alleles (PSA) between a female brown bear and her potential partner (controlled for relatedness and body size) on male mating success estimated using general mixed model implemented in MCMCglmm (see methods for details). Table S2, The effect of AA distance between a female brown bear and her potential partner (controlled for relatedness and body size) on male mating success, estimated using general mixed model implemented in MCMCglmm (see methods for details). Table S3, The association between the number of alleles carried by a female brown bear and the relative number of alleles carried by her mate (n alleles) or relative genetic similarity, measured as proportion of shared alleles (PSA) or amino-acid distance (see Methods for details). Table S4, The effect of the number of MHC alleles carried by a male (controlled for relatedness and body size) on male mating success estimated using general mixed model implemented in MCMCglmm (see methods for details). Removing quadratic term did not change conclusions.
(DOC)
