# Supplementary material for: No Evidence for the Effect of MHC on Male Mating Success in the Brown Bear
Source: PLoS One. 2014 Dec 3;9(12):e113414. doi: 10.1371/journal.pone.0113414 (PMC4254848; doi:10.1371/journal.pone.0113414)
Supplement: File S1 — Supporting files. Table S1, The effect the proportion of shared alleles (PSA) between a female brown bear and her potential partner (controlled for relatedness and body size) on male mating success estimated using general mixed model implemented in MCMCglmm (see methods for details). Table S2, The effect of AA distance between a female brown bear and her potential partner (controlled for relatedness and body size) on male mating success, estimated using general mixed model implemented in MCMCglmm (see methods for details). Table S3, The association between the number of alleles carried by a female brown bear and the relative number of alleles carried by her mate (n alleles) or relative genetic similarity, measured as proportion of shared alleles (PSA) or amino-acid distance (see Methods for details). Table S4, The effect of the number of MHC alleles carried by a male (controlled for relatedness and body size) on male mating success estimated using general mixed model implemented in MCMCglmm (see methods for details). Removing quadratic term did not change conclusions. (DOC) [file pone.0113414.s001.doc]

Table S1. The effect the proportion of shared alleles (PSA) between a female brown bear and her potential partner (controlled for relatedness and body size) on male mating success estimated using general mixed model implemented in MCMCglmm (see methods for details).

|  |  | **Posterior mean** | **Lower 95%CI** | **upper 95% CI** | **Effective sample** | **P_MCMC_** |
| --- | --- | --- | --- | --- | --- | --- |
| **MHC I** | **intercept** | -2.774 | -4.328 | -1.659 | 10.91 | <0.001 |
|  | **PSA** | -0.822 | -6.130 | 4.261 | 79.53 | 0.764 |
|  | **relatedness** | 0.147 | -1.386 | 1.571 | 63.60 | 0.824 |
|  | **body size** | 0.065 | -0.089 | 0.205 | 104.08 | 0.364 |
|  |  |  |  |  |  |  |
| **DRB** | **intercept** | -5.480 | -10.101 | -1.761 | 1.976 | <0.001 |
|  | **PSA** | 3.698 | 14.032 | 3.556 | 62.224 | 0.324 |
|  | **relatedness** | 0.697 | -2.205 | 4.282 | 144.841 | 0.632 |
|  | **body size** | 0.129 | -0.213 | 0.463 | 108.094 | 0.348 |

Table S2. The effect of AA distance between a female brown bear and her potential partner (controlled for relatedness and body size) on male mating success, estimated using general mixed model implemented in MCMCglmm (see methods for details).

|  |  | **Posterior mean** | **Lower 95%CI** | **upper 95% CI** | **Effective sample** | **P_MCMC_** |
| --- | --- | --- | --- | --- | --- | --- |
| **MHC I** | **intercept** | -162.726 | -428.360 | 9.057 | 8.657 | 0.020 |
|  | **AA distance** | 54.486 | -1041.516 | 1192.325 | 195.895 | 0.864 |
|  | **relatedness** | 6.276 | -92.591 | 98.774 | 95.780 | 0.844 |
|  | **body size** | 3.324 | -5.910 | 12.797 | 21.773 | 0.356 |
|  |  |  |  |  |  |  |
| **DRB** | **intercept** | -4.155 | -6.491 | -2.107 | 91.00 | <0.001 |
|  | **AA distance** | 8.985 | -3.764 | 21.879 | 125.62 | 0.172 |
|  | **relatedness** | 0.496 | -0.684 | 1.749 | 83.95 | 0.436 |
|  | **body size** | 0.063 | -0.059 | 0.193 | 161.88 | 0.322 |

Table S3. The association between the number of alleles carried by a female brown bear and the relative number of alleles carried by her mate (n alleles) or relative genetic similarity, measured as proportion of shared alleles (PSA) or amino-acid distance (see Methods for details)

|  |  | **Posterior mean** | **Lower 95%CI** | **upper 95% CI** | **Effective sample** | **P_MCMC_** |
| --- | --- | --- | --- | --- | --- | --- |
| **MHC I** | **n alleles** | -0.0435 | 0.304 | 0.265 | 1000 | 0.746 |
|  | **PSA** | 0.0097 | -0.012 | 0.029 | 1000 | 0.368 |
|  | **AA distance** | -0.002 | -0.007 | 0.002 | 1000 | 0.304 |
|  |  |  |  |  |  |  |
|  | **n alleles** | 0.247 | -0.043 | 0.493 | 884.1 | 0.084 |
| **DRB** | **PSA** | 1.582e-05 | -3.418e-02 | 3.676e-02 | 863.3 | 0.976 |
|  | **AA distance** | 0.0003 | -0.008 | 0.008 | 1000 | 0.934 |

Table S4. The effect of the number of MHC alleles carried by a male (controlled for relatedness and body size) on male mating success estimated using general mixed model implemented in MCMCglmm (see methods for details). Removing quadratic term did not change conclusions.

|  |  | **Posterior mean** | **Lower 95%CI** | **upper 95% CI** | **Effective sample** | **P_MCMC_** |
| --- | --- | --- | --- | --- | --- | --- |
| **MHC I** | **intercept** | -27.154 | -48.707 | -8.6581 | 10.17 | <0.001 |
|  | **n alleles** | 7.732 | -48.301 | 68.761 | 206.42 | 0.796 |
|  | **n alleles^2^** | -5.988 | -61.070 | 49.585 | 328.37 | 0.782 |
|  | **heterozygosity** | 0.206 | -0.006 | 0.436 | 34.66 | 0.026 |
|  | **body size** | 0.306 | -0.294 | 1.065 | 129.00 | 0.326 |
|  |  |  |  |  |  |  |
| **DRB** | **intercept** | -63.807 | -114.157 | -13.719 | 4.865 | <0.001 |
|  | **n alleles** | 34.036 | -99.887 | 171.736 | 262.888 | 0.528 |
|  | **n alleles^2^** | -72.713 | -208.198 | 62.678 | 132.324 | 0.200 |
|  | **heterozygosity** | 0.499 | 0.050 | 1.096 | 13.669 | 0.024 |
|  | **body size** | 0.876 | -0.441 | 2.501 | 100.736 | 0.160 |
